# Effect of Micronutrient and Probiotic Fortified Yogurt on Immune-Function of Anti-Retroviral Therapy Naive HIV Patients 

**DOI:** 10.3390/nu3100897

**Published:** 2011-10-21

**Authors:** Ruben Hummelen, Jaimie Hemsworth, John Changalucha, Nicodemus L. Butamanya, Sharareh Hekmat, J. Dik F. Habbema, Gregor Reid

**Affiliations:** 1 Department of Public Health, Erasmus MC University Medical Center Rotterdam P.O. Box 2040, 3000 CA Rotterdam, The Netherlands; Email: j.d.f.habbema@erasmusmc.nl; 2 Canadian Research & Development Centre for Probiotics, Lawson Health Research Institute, 268 Grosvenor Street, N6A 4V2, London, Ontario, Canada; Email: hekmat@uwo.ca (S.H.); gregor@uwo.ca (G.R.); 3 Division of Food and Nutritional Sciences at Brescia University College, The University of Western Ontario, N6G 1H2, 1285 Western Road, London, Ontario, Canada; 4 National Institute for Medical Research, Mwanza Research Centre, P.O. Box 1462, Mwanza, Tanzania; Email: jchangalucha@yahoo.com; 5 Sekou-Toure Regional Hospital, P.O. Box 132, Mwanza, Tanzania; Email: nicobutamanya@yahoo.com; 6 Departments of Microbiology, Immunology and Surgery, The University of Western Ontario, N6A 5C1, London, Ontario, Canada

**Keywords:** micronutrients, probiotics, HIV, *Lactobacillus*, immune system

## Abstract

Background: Micronutrient supplementation has been shown to reduce the progression of HIV but does not have an effect on the intestinal barrier or the intestinal microbiota of HIV patients. Studies have suggested that probiotics could potentially complement micronutrients in preserving the immune-function of HIV patients. Objective: Assess the impact of micronutrient supplemented probiotic yogurt on the immune function of HIV patients. Design:We performed a randomized, double blind, controlled trial with CD4 count as primary outcome among HIV patients naïve to anti-retroviral treatment. Secondary outcomes included hematological parameters, incidence of diarrhea and clinical symptoms. A total of 112 HIV patients were randomized to receive a micronutrient fortified yogurt with (*n* = 55) or without additional probiotic *Lactobacillus rhamnosus* GR-1 (*n* = 57) for four weeks. Results:An average decline in CD4 count of −70 cells/μL (95% CI: −154 to −15) was observed in the micronutrient, probiotic group *versus* a decrease of −63 cells/μL (95% CI: −157 to −30) in the micronutrient control group (*p* = 0.9). Additional probiotic supplementation was well tolerated and not associated with adverse events. No difference between groups was detected in incidence of diarrhea or clinical symptoms. An improvement of hemoglobin levels was observed for all subjects, based upon a mean difference from baseline of 1.4 g/L (SD = 6) (*p* = 0.02). Conclusion:The addition of probiotics to a micronutrient fortified yogurt was well tolerated by HIV patients but was not associated with a further increase in CD4 count after one month.

## 1. Introduction

Poor nutritional status among people living with HIV is a common problem [1] and has been associated with enhanced HIV progression in numerous observational studies [2,3]. Impaired immune function of people living with HIV is further compounded by a deleterious impact of the virus on the gut-associated mucosal immune system [4,5], which presumably results in translocation of microbial products from the intestinal tract [6,7], an aberrant intestinal microbiota [8,9], and an increased incidence of intestinal infections [10]. Microbial translocation as a result of impaired gut barrier function, in combination with low micronutrient levels, may further enhance the progression of HIV [6,11,12].

Supplementation of micronutrients, most notably B-complex, vitamin C and E, can reduce the progression and mortality of HIV [13,14,15,16,17], but does not impact intestinal barrier impairment [18]. Supplementation of vitamin A as single supplement does not appear to reduce the progression of HIV or mortality related to the disease in adults [15,19].

Probiotics, defined as “live microorganisms which, when administered in adequate amounts, confer a health benefit on the host” [20], have been used with some success to preserve the immune-function of people living with HIV in the following studies. A small randomized controlled trial (RCT) was performed in Brazil among 77 children infected with HIV. Half of the children received a supplement with probiotic strain *Bifidobacterium bifidum *and *Streptococcus thermophilus *for two months. After the two month period, CD4 counts among the treatment group increased 118 cells/μL *versus *a decrease of 42 cells/μL among the placebo group (*p* = 0.05) [21]. Another small pilot RCT in Nigeria discovered a small, but statistically significant effect, of four weeks of *Lactobacillus rhamonus *GR-1 and *Lactobacillus reuteri *RC-14 supplementation on the CD4 count of 24 HIV infected women. The treatment group showed an increase in CD4 of 6.7 cells/μL compared to a decrease of 2.2 cells/μL in the control group (*p* = 0.05) [22]. Lastly, an observational study in Tanzania found that yogurt supplemented with *Lactobacillus rhamnosus *GR-1 increased the CD4 count with 0.28 cells/μL/day (*p* = 0.003) among HIV infected men and women in Tanzania [23]. The mechanisms are believed to involve enhancing the gut barrier function [24,25], alleviating systemic inflammation [26,27], or reducing the duration of gastro-intestinal infections [28] (reviewed in [29]). 

Despite massive efforts, only one out of three people in need of anti-retroviral treatment (ART) currently have access to the treatment [30]. The application of a food-based intervention, targeting both micronutrient deficiencies and an impaired gut-associated mucosal immune system, could provide an optimal adjunctive intervention to potentially delay the progression of HIV. Since 2005, a small community kitchen in Tanzania has produced yogurt supplemented with probiotics using *Lactobacillus rhamnosus* GR-1 [31]. We initiated a randomized, double-blind, placebo controlled study to assess whether the addition of probiotic *L. rhamnosus* GR-1 to a micronutrient fortified yogurt could positively impact the immune function of HIV patients. We selected *L. rhamnosus* GR-1 for its potential to reduce the translocation of *Salmonella typhimurium* from the gut to distant organs, as assessed in a mouse model [32], reduce systemic inflammation in a population with inflammatory bowel disease [33], and to increase the CD4 count in an HIV infected population [23].

## 2. Experimental Section

### 2.1. Study Design

The primary outcome was the mean change of CD4 count from baseline to 4 weeks follow-up. Secondary outcomes included hematology indicators (creatinine, albumin, alanine transaminase (ALT), and full blood count), incidence of diarrheal episodes, symptoms, physical energy and their ability to perform activities of daily living. Inclusion criteria were; confirmed HIV infection, naïve to ART and not eligible for ART initiation (*i.e.*, CD4 count ≥200 with WHO stage I or II infection or CD4 count ≥350 and CDC stage III infection [34]), ability to attend the clinic every other day, ≥18 years of age, not pregnant or lactating, and with no known lactose intolerance or milk allergy. We calculated a sample size of 49 per treatment arm to detect a within person difference of 15 cells/μL in CD4 count between the groups with a power of 90%. The calculation assumed an attrition rate of 25% and a standard deviation of 20 cells/μL within-person change in CD4 count. The Medical Research Coordinating Committee of the National Institute for Medical Research, Tanzania, approved the study design and protocol. Participants were informed of the purpose of the trial and gave their signed or thumb printed informed consent before participation. All study procedures were in concordance with the declaration of Helsinki of 1975, as revised in 2008. 

### 2.2. Study Subjects and Data Collection

Recruitment took place at Shaloom health centre and at Sekou-Toure Regional Hospital, Mwanza, Tanzania from June to July 2008. Randomization and follow-up were conducted at Sekou-Toure regional hospital and extended until August 2008. During the baseline visit, a physical examination was performed by a physician, blood was taken to perform biochemical and hematological measurements and a structured interview was performed on demographics, medical history, and physical energy levels. The participants recorded the occurrence of diarrheal episodes using a diary, which was collected at each visit. Diarrhea was defined as three or more loose or watery stools in a 24-h period [35]. 

Participants were randomized to receive a yogurt that was fortified solely with micronutrients (control group) or was fortified with both micronutrients and *L. rhamnosus *GR-1 (probiotic group) for 4 weeks. Both yogurts were indistinguishable in taste or appearance. Subjects were consecutively assigned to one of the two treatment codes according to a computer generated schedule in blocks of 10. The batches of yogurt were coded by a researcher not involved with data collection to ensure that neither the researchers nor the subjects were aware of their treatment assignments. Participants were asked to return to the clinic every other day to receive a two-day supply of yogurt of which one portion was consumed in the clinic to ensure at least 50% adherence. Subjects returned after 4 weeks for the follow-up visit and were encouraged throughout the duration of the study to report any adverse events or changes in their condition. The procedures of the baseline visit were repeated exactly at the follow-up visit, with additional questions on adverse events. Participants who did not arrive at their follow-up visit were contacted by phone.

### 2.3. Intervention

The nutrient premix blend was provided by Fortitech Strategic Nutrition (Schenectady, New York, NY, USA), for which the concentrations of nutrients per portion of yogurt (125 mL) are summarized in Table 1. We based the formulation on that described by Kaiser *et al*. [17], but reduced the concentrations of the micronutrients to acceptable physiological levels that were also compatible with inclusion into yogurt. Preparation of the probiotic stock culture took place at National Institute for Medical Research (NIMR), Mwanza, and the organism was added to yogurt produced by a trained group of local women. The viability and abundance of the probiotic strain in the yogurt was assured for every batch using procedures previously described [36]. Overall, a mean 1.23 × 10^9^ colony forming units/mL of *L. rhamnosus* GR-1 was found in the probiotic yogurt resulting in an intended dose of 15.38 × 10^10^ colony forming units/day.

### 2.4. Laboratory Measurements

The laboratory measurements were performed at the NIMR, according to good laboratory practices. At baseline and at 4 weeks the CD4 count was measured using the Partec FACS (Partec GmbH, Münster, Germany), hematological parameters were measured using the Beckman Coulter AcT5 Diff Al (Beckman Coulter, Brea, CA, USA) and biochemistry parameters using the Synchron CX^®^4 PRO (Beckman Coulter, Brea, CA, USA). 

### 2.5. Analyses

All analyses were performed on an intent-to-treat basis. The plan of data analysis was clearly established and stated in the study protocol before start of the study. Normally-distributed continuous (CD4 count, hematology parameters) data were compared using within-subject differences from baseline to follow-up, and tested using an unpaired students *t*-test. To assess the effect of the CD4 count, HIV symptoms, BMI and anemia at baseline on the treatment response, a linear regression model was fitted. Categorical data (baseline characteristics) were compared using a χ^2^ test. All tests were performed two-sided at the α = 0.05 level with no adjustments made for multiple comparisons. Compliance with the study treatment was calculated as the number of days the participant consumed the yogurt divided by the total number of days the yogurt should have been consumed. Data was stored in an Access database and analyzed using SPSS 15.0 software.

**Table 1:** Probiotic and micronutrient content of the yogurts.

Ingredient	Amount/125 g
*Lactobacillus rhamnosus* GR-1 *	10^9^ CFU/mL
Vitamin A (as Beta carotene and Palmitate)	1500 IU
Vitamin E (as acetate)	5.7 IU
Niacinamide	3.8 mg
Vitamin B1 (thiamin)	0.3 mg
Vitamin B12 (cyanocobalamin)	0.6 μg
Vitamin B6 (pyroxine)	0.3 mg
Vitamin C (ascorbic acid)	21 mg
Iron (as ferric pyrophosphate)	3.3 mg
Selenium (sodium selenite)	13.8 μg
Zinc (zinc sulphate)	2.4 mg
DHA (omega-3 from fish oil)	13 mg
EPA (omega-3 from fish oil)	19 mg

CFU = Colony forming units; * Only in probiotic-supplemented yogurt.

## 3. Results

### 3.1. Baseline Characteristics

**Figure 1:**
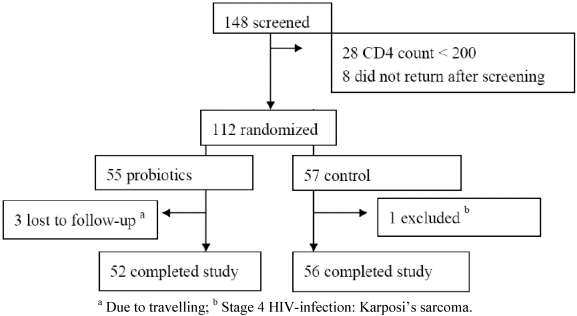
Trial profile.

A total of 148 patients were screened for participation and 112 patients were enrolled. After randomization one participant in the control group was diagnosed with Kaposi’s sarcoma and excluded from analyses according to exclusion criteria, as this indicated that a WHO stage IV had been present at baseline. Of 55 participants in the probiotic group, 52 completed follow-up *versus* all 56 participants in the control group (*p* = 0.1) (Figure 1). At baseline, 21 out of 53 (40%) in the probiotic group had HIV-related symptoms upon physical examination (oral thrush, oral ulcers or maculo-papular rash), compared to 12 out of 55 (21%) in the control group (*p* = 0.03). There was no difference in the median time living with HIV between the probiotic group (791 days, range 57-3511) and the placebo group (584 days, range 31-3458) (Wilcoxon rank test, *p* = 0.2). Other baseline characteristics were comparable between the groups (Table 2). Overall, 92 out of 109 (84%) participants had low serum albumin (≤36 g/L) and 43 out of 97 tested (44%) had anemia at baseline (hemoglobin ≤110 g/L) (Table 3). Compliance was excellent, among the probiotic group the product was consumed at a mean 98% (95% CI: 97-100) of days required compared to 96% (95% CI: 93-100) among the control group (*p* = 0.2). 

**Table 2:** Baseline characteristics of the treatment groups. In a few cases, data were not obtained.

Characteristic ^a^	Reference	Probiotics *n* = 55	Control *n* = 56	*P*
Gender female		47 (86%)	48 (86%)	1.0
Age (years)	<30	10 (19%)	8 (15%)	0.6
	≥30	44 (81%)	46 (85%)	
Water source	Pipe	45 (83%)	41 (73%)	0.2
	Well	9 (17%)	15 (27%)	
Working ability	Unable	5 (10%)	5 (9%)	1.0
	Able	47 (90%)	51 (91%)	
Marital status ^b^	Widow	18 (33%)	24 (43%)	0.8
	Divorced	12 (22%)	16 (28%)	
	Married	19 (34%)	15 (27%)	
	Single	6 (11%)	1 (2%)	
Diagnosed with HIV (years)	<1	18 (33%)	22 (39%)	0.5
	≥1	37 (67%)	34 (61%)	
CD4 Count (cells/mm^3^)	<350	26 (47%)	30 (54%)	0.5
	351-500	12 (22%)	14 (25%)	
	≥501	17 (31%)	12 (21%)	
WHO disease stage ^b^	1	39 (75%)	45 (80%)	0.5
	2	5 (10%)	4 (7%)	
	3	8 (15%)	7 (13%)	
Any HIV related symptom ^c^		21 (38%)	12 (21%)	0.03
BMI	<18.5 kg/m^2^	1 (2%)	6 (11%)	0.1
Anemia (Hemoglobin)	<110 g/L	22 (40%)^ d^	21 (38%)	0.6
Hypoalbuminemia (Albumin)	<36 g/L	43 (78%)^ e^	49 (88%)	0.4
Clotrimoxazole use		16 (29%)	12 (21%)	0.4
Vitamin B complex use		6 (11%)	4 (7%)	0.5

BMI = Body Mass Index. ^a^ The following categories have missing values: Age (3), water source (1), Functional Status (3), BMI (4), WHO disease stage (3), Any HIV related symptom (3). ^b^ The following subgroups were combined for valid testing: Marital status: “Widow” with “divorced” with “single”; WHO disease stage: stage “2” with “3”; ^c^ Any HIV related symptom = oral thrush, oral ulcers or maculo-papular rash on physical examination. As not all data could be obtained, some of the calculations in the table are not for the full 55 or 56 participatants; ^d^ there were missing biological samples for 8 of the participants at baseline in the probiotic group, *i.e.*,22 of 47 showed low hemoglobin levels in the probiotic group; ^e^ there were two missing samples for albumin in the probiotic group.

**Table 3:** Changes in hematology and biochemistry parameters from baseline to follow-up.

		Probiotics				Control		
Parameter	Reference	Base ^a^	FU	% Change ^b^	Base	FU	% Change	*p*^c^
	Value/unit	n/total	n/total	mean ± SD	n/total	n/total	mean ± SD	
Creatinine	≥139 μmol/L	1/52	0/50	−1 ± 17	0/56	1/56	1 ± 29	0.6
Albumin	<36 g/L	43/53	46/50	0 ± 20	49/56	51/56	−1 ± 24	0.9
ALT	≥50 IU/L	2/53	2/50	−3 ± 9	2/56	1/56	1 ± 10	0.1
Hemoglobin	<110 g/L	22/47	19/43	−9 ± 53	21/50	17/47	4 ± 53	0.3
Platelets	<150 × 10^9^/L	3/47	3/43	−13 ± 47	4/50	4/47	−5 ± 23	0.3
RBC	<4 × 10^12^/L	29/47	17/43	−10 ± 53	33/50	14/47	−6 ± 66	0.7
WBC	≥11 × 10^9^/L	0/48	0/43	−1 ± 18	1/50	0/47	−7 ± 21	0.2
Neutrophils	≥80%	0/44	0/37	−6 ± 38	0/46	0/45	−12 ± 72	0.6
Lymphocytes	≥50%	13/47	13/43	−6 ± 36	11/50	14/47	10 ± 72	0.2
Monocytes	≥10%	0/47	0/43	1 ± 12	0/50	0/47	−1 ± 15	0.6
Eosinophils	≥5%	23/44	19/37	−7 ± 16	26/46	28/45	−1 ± 12	0.1
Basophils	≥2%	0/47	0/43	−2 ± 4	0/50	0/47	−1 ± 4	0.1

Base = Baseline; FU = Follow-up; SD = Standard Deviation; ALT = Alanine transaminase; RBC = Red blood cells; WBC = White blood cells; ^a^ Number of participants above or below reference value; ^b^ Mean within-subject change from baseline to follow-up; ^c^ Tested using within-subject change from baseline to follow-up.

### 3.2. Primary Outcome

The probiotic group experienced an average decline of −70 cells/μL (95% CI: −154 to −15 cells/μL) CD4 cells from baseline *versus* a decline of −63 cells/μL (95% CI: −157 to −30) in the control group (*p* = 0.9). Adjusting for baseline CD4 count, HIV symptoms, BMI or anemia yielded similar results and did not change this finding. The baseline CD4 count was highly predictive of the development of the CD4 count during follow-up. Overall, those with a CD4 count of 200-350 experienced an increase in CD4 count of 67 cells/μL (95% CI: −19 to 152) during follow-up. This in contrast to those with a CD4 count of ≥350 cells/μL at baseline who experience an average decline of 187 cells/μL (95% CI: −372 to −136) during follow-up. Among those with a CD4 count of 200-350 cells/μL, HIV related symptoms were more common (21/53) than those with a CD4 count of ≥350 cells/μL (12/55) (*p* = 0.05). No differences were detected in the number of days living with HIV between those with a CD4 count of 200-350 or ≥350 cells/μL at baseline.

### 3.3. Secondary Outcomes

No differences were found in self-reported symptoms (mouth ulcers, coughing, fever, nausea, and stomach pain), self-reported physical energy level or ability to perform daily activities following the one month treatment. Among the probiotic group, 13 out of 51 (26%) participants experienced diarrhea for at least one day during the intervention, which was not different from the control group, where 16 out of 54 (30%) experienced diarrhea (*p* = 0.6). Overall, an improvement of hemoglobin levels was observed with a mean difference from baseline of 1.4 g/L (SD = 6) (*p* = 0.02).

### 3.4. Adverse Events

In the probiotic group, four participants reported an adverse event including one with stomach pain, two with nausea and one with diarrhea as the main adverse event. In the control group, six participants reported an adverse event, among which one subject reported numbness in hands and feet, one stomach pain and three reported having diarrhea. Only the numbness of hands and feet symptoms were scored severe by a physician but this was unlikely to be related to the study intervention. The other adverse events were rated mild or moderately. No differences were detected in biochemical or hematological parameters between the probiotic and control group (Table 3). 

## 4. Discussion

In this randomized, controlled study, the addition of a probiotic *Lactobacillus* to a micronutrient fortified yogurt did not further substantiate the effect on the CD4 count or clinical symptoms of HIV-infected adults with a starting CD4 count of ≥200 cells/μL. The micronutrient fortified probiotic yogurt was well tolerated among the participants and was not associated with adverse events. This result somewhat contrasts previous studies showing a positive effect of probiotic supplementation on CD4 counts [21,22,23], but these were not performed with micronutrient supplementation which herein and elsewhere [17] independently can affect on CD4 count. One small RCT in Nigeria had used *L. reuteri* RC-14 in combination with the strain studied here (*L. rhamnosus* GR-1) [22], while the observational study had used *L. rhamnosus* GR-1 alone [23]. The RCT in Brazil had used a combination of different strains for children treated with ART (*Bifidobacterium bifidum *and *Streptococcus thermophilus*) [21]. The apparent contradiction could have two explanations. Firstly, the study in Nigeria [22] had found a standard deviation of the change in CD4 count of approximately 10 cells/μL during a 4 week follow-up while in our population we found a standard 330 cells/μL during the same follow-up period. Because we used the previously reported standard deviation for sample size calculations, our study had reduced power to show an effect. The Nigerian study had recruited a homogenous group of women having moderate diarrhea symptoms, which could explain the difference in variability in CD4 counts. Secondly, our treatment period could have been too short to show an effect on CD4 count. Upon reflection, the use of measurements of the intestinal barrier function (e.g., lipo-polysaccharide or lactoferrin: mannitol ratio [6,7]), or immune activation and regulation (e.g., CD4+/CD25+ and FoxP3 lymphocytes [30,37]) could have served as a screening tool to assess potential benefits of probiotics within an HIV infected population. Therefore, we suggest these markers to be used in future studies to screen or assess the potential effect of specific probiotic strains.

The overall change in CD4 count of a mean −70 cells/μL (CI 95%; −157 to −30) was lower than normally observed among HIV patients [38,39]. The decrease among both groups was not expected as the micronutrient formula tested by Kaiser *et al. *[17], on which our micronutrient formula was based, had shown an increase in CD4 count. The nutrient formula used in this study was not intended to provide a high dose of micronutrients at multiple times of the Dietary Reference Intake (DRI) [15,17], but rather an excellent dietary source at 25% of the DRI [40] to an already nutritious food (yogurt). The decrease in CD4 count may be explained by a tendency of parameters to decline when selected above a specific value (*i.e*., regression to the mean). Since HIV patients were included only with a CD4 count ≥200 cells/μL, a greater decline in CD4 count could have been observed than within an unselected population. This explanation is supported by the finding that participants with a CD4 count ≥350 cells/μL at baseline experienced decrease (−187 cells/μL) while those with a CD4 count of <350 cells/μL experience an increase in CD4 count (67 cells/μL). The impact of this mechanism on the results was assessed by adjusting for baseline CD4 count levels; this adjustment did not significantly change the inferences of the study, and is therefore likely to be modest. The decrease might further be explained in part by the fact that almost half of the participants had anemia, which has been observed to be factors for enhanced HIV progression [41]. Again, it is easy in hindsight to consider how better the study could have been set up, but clearly lessons have been learned that even with limited resources, attempts to identify potential responders to the treatment should be made [41]. In addition, future studies of longer duration should be performed to assess whether benefits take longer to accrue. 

## 5. Conclusions

In conclusion, the addition of a probiotic strain to a yogurt already supplemented with micronutrients was well tolerated by people living with HIV with a CD4 count ≥200 cells/μL, but was not associated with further preservation of the immune-function over a one month period. The fact that a yogurt could be made in a community kitchen that is acceptable in terms of taste and texture, and contains a complex mixture of nutritional supplements, offers some hope that grass root, nutrition-based approaches can potentially be found that contribute to the quality of life of people living with HIV.
